# New insights into Fe localization in plant tissues

**DOI:** 10.3389/fpls.2013.00350

**Published:** 2013-09-06

**Authors:** Hannetz Roschzttardtz, Geneviève Conéjéro, Fanchon Divol, Carine Alcon, Jean-Luc Verdeil, Catherine Curie, Stéphane Mari

**Affiliations:** Biochimie et Physiologie Moléculaire des Plantes, Centre National de la Recherche Scientifique, Institut National pour la Recherche Agronomique, Laboratoire de Biochimie et Physiologie Moléculaire des Plantes, INRA/SupAgro,Université Montpellier 2Montpellier, France

**Keywords:** iron, Arabidopsis, root, chloroplast, ferritin, pollen, amyloplast, mitochondria

## Abstract

Deciphering cellular iron (Fe) homeostasis requires having access to both quantitative and qualitative information on the subcellular pools of Fe in tissues and their dynamics within the cells. We have taken advantage of the Perls/DAB Fe staining procedure to perform a systematic analysis of Fe distribution in roots, leaves and reproductive organs of the model plant *Arabidopsis thaliana*, using wild-type and mutant genotypes affected in iron transport and storage. Roots of soil-grown plants accumulate iron in the apoplast of the central cylinder, a pattern that is strongly intensified when the citrate effluxer FRD3 is not functional, thus stressing the importance of citrate in the apoplastic movement of Fe. In leaves, Fe level is low and only detected in and around vascular tissues. In contrast, Fe staining in leaves of iron-treated plants extends in the surrounding mesophyll cells where Fe deposits, likely corresponding to Fe-ferritin complexes, accumulate in the chloroplasts. The loss of ferritins in the *fer1,3,4* triple mutant provoked a massive accumulation of Fe in the apoplastic space, suggesting that in the absence of iron buffering in the chloroplast, cells activate iron efflux and/or repress iron influx to limit the amount of iron in the cell. In flowers, Perls/DAB staining has revealed a major sink for Fe in the anthers. In particular, developing pollen grains accumulate detectable amounts of Fe in small-size intracellular bodies that aggregate around the vegetative nucleus at the binuclear stage and that were identified as amyloplasts. In conclusion, using the Perls/DAB procedure combined to selected mutant genotypes, this study has established a reliable atlas of Fe distribution in the main Arabidopsis organs, proving and refining long-assumed intracellular locations and uncovering new ones. This “iron map” of Arabidopsis will serve as a basis for future studies of possible actors of iron movement in plant tissues and cell compartments.

## Introduction

Iron (Fe) is an essential metal that plays a central role in many cellular mechanisms. The transition between two redox states, ferrous and ferric iron, involving the gain or loss of one electron, is a key feature for a wide variety of reactions requiring Fe, such as the electron transport chains of the photosynthesis or respiration, the synthesis of nucleotides and chlorophyll. Plants, as sessile organisms, have to continuously adapt to changing conditions. Regarding Fe, dicotyledonous plants such as *Arabidopsis thaliana* have developed efficient strategies to acquire Fe from the soil, where the availability of this metal is often extremely low, by the expression of the root ferric chelate reductase encoded by FRO2 and the Fe^2+^ transporter encoded by IRT1 (Eide et al., 1996; Robinson et al., 1999; Vert et al., 2002). In the meantime, Fe excess can be harmful and induce oxidative stress due to the high reactivity of Fe^2+^ with O_2_ to produce reactive oxygen species. When challenged with high Fe concentrations, plants induce the expression of ferritins (Lobreaux et al., 1992). Ferritins are plastidial proteins with the capacity of complexing several thousands of Fe atoms when associated in 24-mer multimers. By analogy with animal systems, plant ferritin was thought to play a key role in buffering Fe excess in plants (Briat and Lobreaux, 1998; Briat and Lebrun, 1999). The function of ferritins may be more complex since these proteins have recently been shown to play a more direct role in the protection against oxidative damage (Ravet et al., 2009). Overall, plants have to maintain a strict Fe homeostasis to achieve proper growth and development. This is achieved through the tight regulation of the physiological functions of root absorption, long distance circulation, storage and remobilization.

Many genes involved in Fe homeostasis have been identified by genetic or transcriptomic approaches. Over the last 15 years, a wealth of important advances has been obtained to understand the mechanism of Fe homeostasis, including the identification of molecular actors of Fe transport, circulation and sequestration. On the contrary, the precise localization of the Fe pools as well as the dynamics of these pools at the tissue, cellular and sub-cellular levels remain elusive.

In roots, the apoplast has been proposed to play an important role in the storage of Fe following absorption (Bienfait et al., 1985). Although it has been shown biochemically, by reduction and complexation, that the Fe binding and exchanging capacities of the apoplast can be extremely high, these measurements do not reflect the real quantity of apoplastic Fe found in roots of plants grown in soil (Strasser et al., 1999). Beside the biochemical approach described by Bienfait et al. (1985), Fe can also be detected by histochemical staining with the Perls reagent. Specific for Fe^3+^, the Perls staining procedure was a valuable tool to show that roots of FRD3 mutant plants, impaired in citrate loading in the xylem, accumulated high amounts of Fe in the central cylinder, in both Arabidopsis and rice mutant genotypes (Green and Rogers, 2004; Yokosho et al., 2009). However, the spatial resolution of the Perls images was not high enough to identify Fe location at the cellular or the sub-cellular level in these roots.

In leaves it is predictable that an important portion of Fe will be located in chloroplasts, since a complete electron transfer chain contains 22 atoms of Fe (Wollman et al., 1999). It could thus be expected that Fe would be evenly distributed in the leaf mesophyll tissues. Actually, several studies have reported that Fe is highly concentrated in the vasculature of leaves from Arabidopsis (Stacey et al., 2008), peach-almond hybrids (Jimenez et al., 2009) and tobacco (Takahashi et al., 2003). In contrast, by performing sub-cellular fractionation and organelle purification of Arabidopsis leaves, approximately 70% of the total Fe measured was found in the chloroplastic fraction, of which one half was attributed to the thylakoids (Shikanai et al., 2003). Overall, clear information on the localization of Fe pools in leaves, at the cellular and sub-cellular levels is still missing. The discrepancies between the reports cited above may be due to (i) the complexity of the organ in terms of cell types, and (ii) the technical bias such as the low penetration of Perls in hydrophobic tissues or substantial metal loss during organelle fractionation.

The Arabidopsis embryo has recently emerged as an ideal model to study iron distribution and localization. The three dimensional imaging of metals in seeds, obtained by micro X-ray fluorescence (μXRF) and tomography, beautifully showed the specific accumulation of Fe around the pro-vascular system of the embryo, whereas manganese (Mn) was concentrated in the abaxial part of the cotyledons and zinc (Zn) uniformly distributed in all embryonic cells (Kim et al., 2006). This pattern of Fe was further shown to depend on the activity of the tonoplastic iron transporter VIT1, since in a *vit1* knockout mutant the vascular distribution was abolished. To reach a sub-cellular resolution, Energy Dispersive X-ray microanalysis (EDX) and inelastic scattered microscopy techniques were used (Lanquar et al., 2005). The authors have identified a Fe pool in globoid structures that are engulfed in the vacuoles (Lanquar et al., 2005). Furthermore, this Fe pool was shown to be remobilized from the vacuoles during germination by the efflux transport activity of the NRAMP3 and NRAMP4 tonoplastic proteins (Lanquar et al., 2005). More recently, we have reported the development of a highly sensitive histochemical staining of Fe that combines Perls staining with a second step of intensification with diamino benzidine (DAB) and H_2_O_2_ (Roschzttardtz et al., 2009). With this simple and quick method (termed Perls/DAB from now on) we have shown that both Fe2+ and Fe3+ could be detected, with a definition and sensitivity comparable to μXRF (Roschzttardtz et al., 2009). The problems of dye penetration in tissues were overcome by applying the staining procedure directly on the histological sections, hence dramatically increasing the image resolution, although in this case the loosely bound iron forms could be washed away and lost during fixation and dehydration steps (Roschzttardtz et al., 2011a).

In Arabidopsis embryos, a detailed histological analysis with Perls/DAB, combined with suited developmental mutants, enabled to establish that the Fe accumulated around the provascular system was in fact located in a single cell layer corresponding to the endodermis. Moreover, we could show that in endodermal cells Fe was located within the vacuoles, most likely in the globoid structures described above. This vacuolar storage of iron is not a general feature of seeds. We have recently shown that in pea embryos an important pool of Fe is actually located in the nucleus, the highest local concentration of Fe being in the nucleolus (Roschzttardtz et al., 2011a). This unexpected discovery could actually be extended to many cell types in different species, uncovering a potential new role of Fe in the nucleolus.

Besides providing basic information on the localization of an essential metal in plant cells, the different reports on embryo Fe localization have illustrated convincingly the pivotal contribution of imaging approaches to the comprehension of the function of genes (VIT1, NRAMP3, NRAMP4) that would not have been possible otherwise. However, basic questions remain unaddressed, such as to what extent apoplastic and vacuolar compartments can buffer Fe, how much Fe is located in plastids and mitochondria, or have other cellular compartments been completely overlooked in terms of Fe storage?

Our previous work has illustrated the power of the Perls/DAB staining method to pinpoint Fe-containing compartments in plant cells. We therefore, sought to extend the use of the Perls/DAB method to the rest of the plant, in order to establish an atlas of Fe distribution and localization in the model plant Arabidopsis. Here we report on Fe distribution in roots, leaves and flowers with a particular emphasis on pollen grains. In order to avoid potential artifacts originating from the source of iron in the growth medium, the plants were only grown on soil. Apart from wild-type plants, two mutants were included in this study, *frd3*, impaired in Fe translocation from roots to shoots and the triple *fer1,3,4* mutant, devoid of ferritin proteins in leaves (Ravet et al., 2008, 2009).

## Materials and methods

### Plant material and growth conditions

*Arabidopsis thaliana* plants were grown on soil (Humin Substrate N2 Neuhaus; Klasmann-Deilmann, Geeste, Germany) in a greenhouse at 23°C. The plants were irrigated with H_2_O or supplemented with iron (irrigation with 2 mM FeEDDHA for 48 h), as indicated in the legend of each Figure. The genotypes used in this study were Col-0 (wild-type), the triple ferritin mutant *atfer1,3,4* (Ravet et al., 2008) and the *frd3-7* mutant (Roschzttardtz et al., 2011b). The *atfer1,3,4* mutant is devoid of ferritin proteins in all plant organs except in seeds and has no macroscopic phenotype in standard conditions, although the mutant plants are more sensitive to oxidative stress (Ravet et al., 2008). The *frd3* mutant, severely impaired in root-to-shoot translocation of Fe, accumulates high amounts of Fe in the stele (Green and Rogers, 2004; Durrett et al., 2007).

### Histochemical staining of Fe with the perls/DAB procedure

For organ staining, the flowers were vacuum infiltrated with equal volumes of 4% (v/v) HCl and 4% (w/v) K-ferrocyanide (Perls stain solution) for 15 min and incubated for 30 min at room temperature (Stacey et al., 2008). The DAB intensification was performed according to Roschzttardtz et al. (2009). After washing with distillated water, the flowers were incubated in a methanol solution containing 0.01 M NaN_3_ and 0.3% (v/v) H_2_O_2_ for 1 h, and then washed with 0.1 M phosphate buffer (pH 7.4). For the intensification reaction the flowers were incubated between 10 and 30 min in a 0.1 M phosphate buffer (pH 7.4) solution containing 0.025% (w/v) DAB (tetrahydrochloride, Sigma, St Louis, Mo, USA), 0.005% (v/v) H_2_O_2_, and 0.005% (w/v) CoCl^*^_2_6H_2_O (intensification solution). Rinsing with distilled water stopped the reaction.

For the *in situ* Perls/DAB/H_2_O_2_ intensification, different organs were vacuum infiltrated with the fixation solution containing 2% (w/v) paraformaldehyde, 1% (v/v) glutaraldehyde, 1% (w/v) caffeine in 100 mM phosphate buffer (pH 7) for 30 min and incubated for 15 h in the same solution. For leaves, the fixation solution alos contained 0.01% (v/v) triton X-100. The roots were previously rinsed with distilled water and traces of soil were eliminated using a binocular magnifying lens. The fixed samples were washed with 0.1 M Na-phosphate buffer (pH 7.4) three times, and dehydrated in successive baths of 50, 70, 90, 95, and 100% Ethanol, butanol/ethanol 1:1 (v/v) and 100% butanol. Then, the tissues were embedded in the Technovit 7100 resin (Kulzer) according to the manufacturer's instructions and thin sections (3 μm) were cut. The sections were deposited on glass slides that were incubated for 45 min in Perls stain solution. The intensification procedure was then applied as described above.

### Ferritin inmunolocalization

The rosette leaves from 3 week-old plants grown in greenhouse were vacuum infiltrated with 4% (w/v) paraformaldehyde in 10 mM PBS pH 7.2 buffer (7 mM NaHPO_4_, 3 mM NaH_2_PO_4_, 120 mM NaCl, 2.7 mM KCl) for 1 h and incubated overnight in the same solution. The samples were washed with 100 mM glycine in 10 mM phosphate buffer (pH 7.5) three times, and dehydrated in successive baths of 50, 70, 95, and 100% ethanol, butanol/ethanol 1:1 (v/v), and 100% butanol. The tissue embedding was performed with successive baths of increasing concentrations of Safesolv (Labonord, France) in butanol and then with Safesolv/Paraplast pura wax (Parrafin X-TRA, McCormick Scientific) baths at increasing pure wax concentration. Paraplast blocks were cut with a razor blade at 8 μm thickness (Leica microtome RM 2265). The cross sections were transferred on silanized slides (DakoCytomation, http://www.dako.com) and completely dried. The samples were then dewaxed and rehydrated following the converse steps. After blocking overnight by incubation with 5% BSA (w/v) in PBS, the samples were treated with trypsin (0.1%) for 10 min at room temperature, and washed in PBS (2 × 5 min). Trypsine activity was inhibited by adding trypsin inhibitor (0.05%) during 5 min at room temperature and washed in PBS (2 × 10 min). After overnight incubation (4°C) with rabbit anti-Ferritin polyclonal antibody (1/500), the sections were washed with PBS (3 × 10 min) and incubated with anti-rabbit IgG F(ab')_2_ fragment conjugated to the Alexa Fluor 488 fluorochrome (Invitrogen) for 1 h at room temperature in the dark. After washing in PBS (3 × 10 min), the sections were incubated with DAPI solution staining during 10 min at room temperature. After washing in PBS (3 × 10 min), the sections were mounted in Mowiol anti-fading medium and kept at 4°C until analyzed.

### Laser scanning confocal microscopy

The microscope imaging was performed in Montpellier RIO Imaging Platform (http://www/mri/cnrs.fr) with a confocal microscope (LSM 510, Meta; Carl Zeiss MicroImaging, http://www.zeiss.de). An Argon laser at 488 nm provided excitation for the Alexa 488, and 405 nm was used for DAPI staining. The fluorescence emission signals were detected in Multi Track using a band-pass filter of 505–530 nm for the Alexa Fluor 488, a long-pass filter of 650 nm for the far-red autofluorescence of the chloroplast and a band-pass filter of 420–480 nm for DAPI. The sections were observed with a x63 oil Zeiss objective. Pictures were processed using the Zeiss LSM Image Browser software.

### Mitochondria staining with DiOC6

Thin sections of resin-embedded anthers (2 μm thick) were stained with 3,3′-Dihexyloxacarbocyanine Iodide (DiOC6) as previously described (Nagata et al., 2000). Sections were incubated with 100 μg of DiOC6 per mL of ethanol for 1 min, rinsed with 50% (v:v) ethanol for 1 min and with distilled water for 1 min. The sections were then mounted in Mowiol anti-fading medium and kept at 4°C until analyzed. Observations were realized with an epifluorescence microscope (Olympus BX61, Ex = 470 nm ± 20, dichroïc mirror 495 nm, Em = 500–530 nm).

### Polysaccharide staining with periodic acid-schiff reagent

Thin sections of resin-embedded anthers (2 μm thick) were incubated 10 min in 1% (w/v) periodic acid solution and then rinsed with distilled water. Subsequently, the sections were stained with Schiff's reagent (Labonord, France) for 20 min and rinsed rapidly once with 0.25% (w/v) sodium metabisulfite, 0.05 N HCl, followed by once with distilled water, and observed under a light microscope.

## Results

### Iron distribution in roots

**Figure 1:**
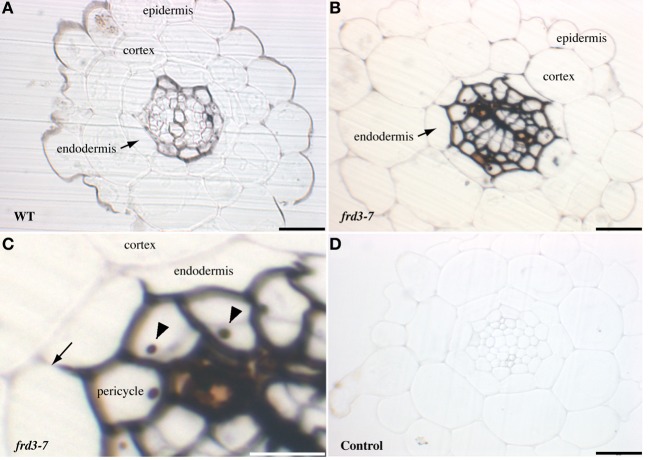
**Iron detection in Arabidopsis roots.** Sections of 4-week old WT **(A)** or *frd3-7* **(B–D)** mutant plants stained with Perls/DAB. Panel **(C)** corresponds to a magnified image of panel **(B)**, both showing iron restriction within the boundary of the Casparian strip (arrow), nucleoli are indicated by arrowheads. **(D)**, control section stained with DAB without previous Perls reaction. Scale bars: 50 μm **(A,B,D)** or 25 μm **(C)**.

Once absorbed in root epidermal cells by the IRT1 ferrous iron transporter, iron must be translocated to the shoot through the vascular system. In order to determine where iron is located during this process, roots of Arabidopsis plants grown in soil were stained with Perls/DAB, which specifically stains iron (Roschzttardtz et al., 2009). Although staining intensity varied from root to root (data not shown) we observed that Fe accumulated in the stele (Figure 1A). The staining appeared consistently higher around the pericycle cells and the xylem tracheary elements. We also used a genetic model for iron accumulation in the root, the *frd3* mutant, which had been reported to accumulate high levels of iron in the root vascular region (Green and Rogers, 2004). FRD3 encodes a membrane protein that controls citrate loading of the xylem (Rogers and Guerinot, 2002; Green and Rogers, 2004; Durrett et al., 2007) To visualize more precisely the impact of the loss of FRD3 on the distribution of iron, root sections of *frd3-7* grown in soil were stained with Perls/DAB. As in WT roots, Fe staining was restricted to the vascular tissue, however, the intensity of the signal was dramatically increased compared to WT (Figures 1B,C). These results are compatible with the current hypothesis that in the absence of citrate efflux activity in the xylem parenchyma cells, iron is prone to precipitation and is retained in the apoplastic compartment. Interestingly, iron staining in *frd3* roots outlined exactly the inner most half of the endodermal cells, thus revealing the role of the casparian strip in restraining apoplastic Fe in the inner layers of the root (Figure 1C). Beside the cell wall compartment, some pericyle cells contained a Fe-rich structure that most likely corresponded to the nucleolus (Figure 1C).

### Iron distribution in leaves

The chloroplast is a well-known sink compartment for iron since each chain of electron transport contains as many as 22 Fe atoms that are used as cofactors. Indeed, iron deficiency impairs photosynthesis and provokes a characteristic chlorosis of the leaves. Moreover, the ubiquitous storage protein Ferritin is located in plastids in plants where it accumulates in response to iron overload. Although it is assumed, for these reasons, that the chloroplast represents an important iron storage compartment in the leaf, little is known about iron localization in this organ.

**Figure 2:**
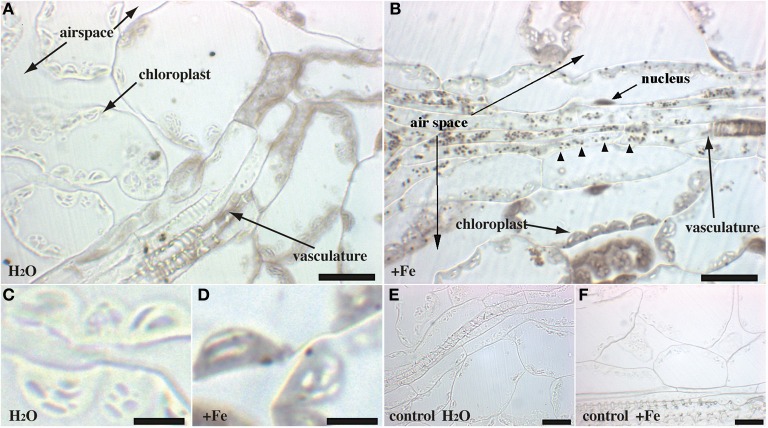
**Iron excess in Arabidopsis rosette leaves.** Sections of 4-week old WT plants irrigated with either water **(A,C,E)** or Fe-EDDHA 2 mM during 48 h **(B,D,F)** were stained with either Perls/DAB **(A–D)** or DAB alone as a negative control **(E,F)**. The panels **(C,D)** correspond to a magnified image of regions of panels **(A,B)**, respectively. Arrowheads in panel **(B)** indicate Fe-rich structures in the vascular tissues. Scale bars: 20 μm **(A,B,E,F)** or 5 μm **(C,D)**.

**Figure 3:**
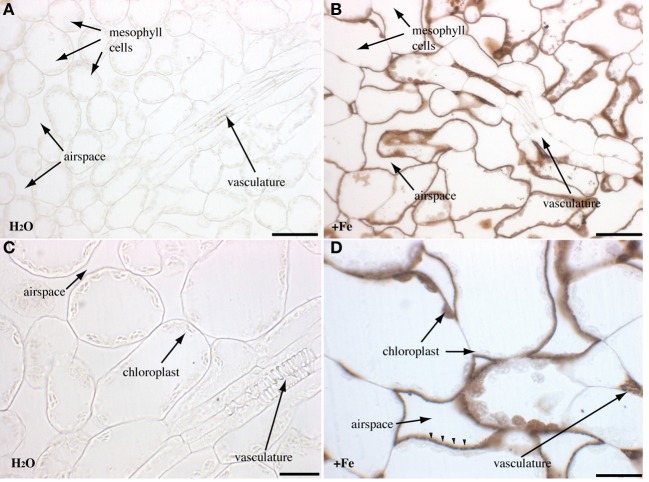
**Iron excess in rosette leaves of the *Atfer134* triple mutant.** Sections of 4-week old *Atfer134* plants irrigated with either water **(A,C)** or Fe-EDDHA 2 mM during 48 h **(B,D)** were stained with either Perls/DAB **(A,B,D)** or DAB alone as a negative control. **(C)** Fe accumulation in the extracellular space is indicated by arrowheads in panel **(D)**. Scale bars: 50 μm **(A,B)** or 20 μm **(C,D)**.

**Figure 4:**
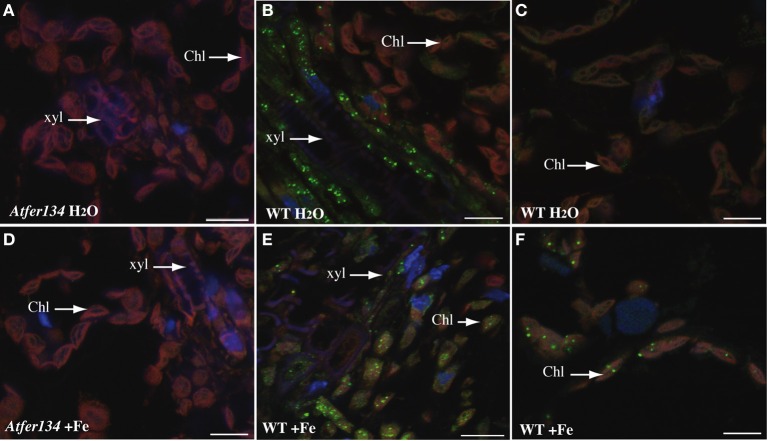
**Immunolocalization of Ferritin in Arabidopsis leaves.** Sections of rosette leaves from 3 week-old WT **(B,C,E,F)** or triple mutant At*fer1,3,4* **(A,D)** plants irrigated with either water **(A–C)** or 2 mM Fe-EDDHA **(D–F)** were probed with an anti-ferritin antibody and revealed with a secondary anti-rabbit antibody coupled to the Alexa Fluor® 488 fluorophore. Sections were stained with DAPI to reveal cell nuclei. Ferritin localization appears in green, DAPI fluorescence in blue and chlorophyll auto-fluorescence in red. Scale bars: 10 μm.

Rosette leaves of 4 week-old plants grown in soil were used to visualize the main iron pools by Perls/DAB staining, either in normal iron (irrigation with water) or in iron excess (irrigation with 2 mM FeEDDHA for 48 h) conditions. Since previous reports indicated that Fe was more concentrated around the veins, we tried to realize longitudinal/tangential sections in order to have more informative pictures of the vascular system and the surrounding cells. All the leaf sections presented (Figures 2–4) correspond to the spongy mesophyll region of the leaves, where round cells are loosely arranged, with numerous air spaces. Leaf thin sections of plants grown on standard Fe exhibited a fuzzy staining in the chloroplasts of some of the mesophyll cells, as well as in the vasculature at the level of the xylem vessels and neighboring parenchyma cells (Figures 2A,C). No staining was detected in the control slide without Perls (Figures 2E,F). Leaves treated with iron showed a stronger staining in the plastids with defined black dots within the organelle indicating the presence of elevated iron concentration (Figures 2B,D). In addition, Fe excess triggers the appearance of numerous dots of Fe in parenchyma cells of the vasculature region (Figure 2B). Because the ferritin protein is accumulated to buffer Fe in excess conditions, we tested whether Fe distribution was altered in the *Atfer134* triple mutant that retains no detectable ferritins in leaves (Ravet et al., 2008). *Atfer134* leaf sections prepared from plants grown on Fe excess showed a large quantity of iron in plastids, compared to optimal Fe plants that did not show detectable staining of Fe (Figures 3A,B). Moreover, the Fe-rich dots observed in wild-type plastids and in the vascular parenchyma were absent in *Atfer1,3,4* (Figures 3B,D). These results strongly suggest that Perls/DAB detects Fe-ferritin in leaves in two locations, in the chloroplast of mesophyll cells and in xylem parenchyma cells in a sub-cellular compartment that remains to be identifed. Interestingly, the *Atfer134* mutant accumulated a dramatic amount of iron in the extracellular space, suggesting a profound effect of the loss of ferritin on iron homeostasis in leaf cells (Figures 3B,D).

To further support the view that Perls/DAB-stained dots in mesophyll chloroplasts and in vasculature cells are Fe-ferritin complexes, we performed inmunolocalization of ferritins in rosette leaves of plants treated or not with FeEDDHA (Figure 4). No signal was detected in *Atfer134* even in iron excess conditions (Figures 4A,D). In wild-type leaves, the ferritin was detected in xylem-associated cells in standard conditions but not in mesophyll cells (Figures 4B,C). In iron excess conditions, however, punctuate labeling appeared in chloroplasts of the mesophyll cells (Figures 4E,F). The presence of ferritin in the vasculature is therefore compatible with the presence of Fe-ferritin in these cells in response to iron excess. Whether ferritins are located in plastids in these cells like in mesophyll cells remains to be established.

### Iron distribution in the flower

**Figure 5:**
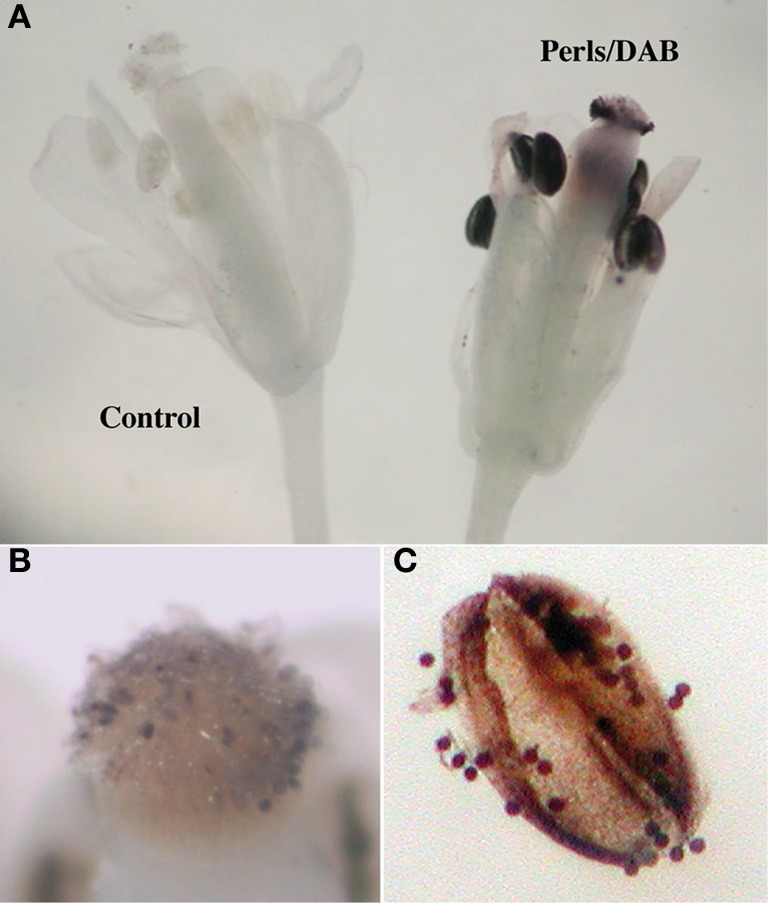
**Arabidopsis flowers stained with Perls/DAB. (A)** Open mature flowers, **(B)** stigmatic papillae with sticking pollen grains, **(C)** anther with mature pollen grains.

Iron accumulation or distribution in flowers is still uncharacterized. However, the importance of iron in flowers is illustrated by the frequent sterility observed in iron homeostasis defective mutants and by the numerous genes encoding iron acquisition components that are expressed in this organ (Stacey et al., 2002; Vert et al., 2002; Connolly et al., 2003; Waters et al., 2006). In order to determine in which tissues and cell compartments iron is accumulated in flowers, entire Arabidopsis flowers were stained with Perls/DAB. A strong black precipitate was observed in anthers and mature pollen grains revealing that the male reproductive organ is an important sink for iron in the plant (Figures 5A,C). The staining observed on top of the pistil is exclusively produced by pollen grains that are attached to the stigma in open flowers (Figure 5B). Thus, the Perls/DAB staining method did not detect iron in floral organs other than the anther, indicating that the anther alone contains large pools of Fe.

**Figure 6:**
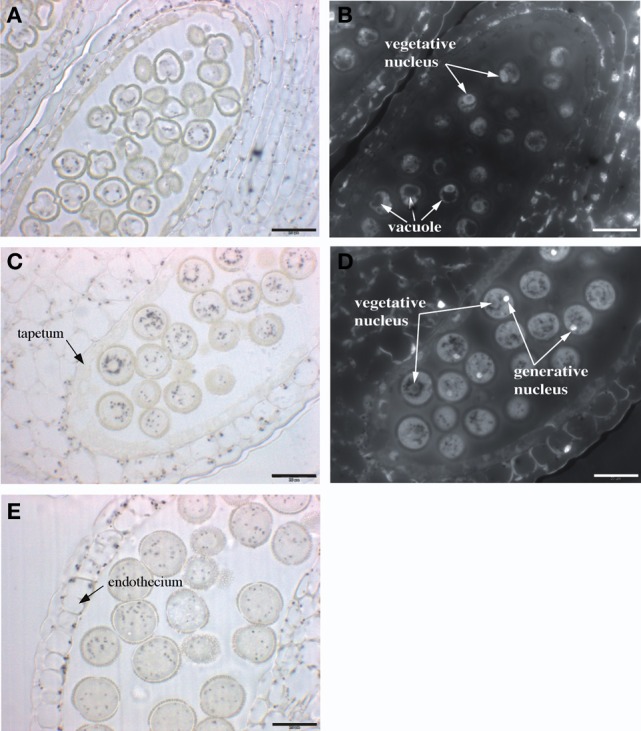
**Iron distribution during Arabidopsis pollen development.** Sections of anthers at three different stages of pollen development were stained with Perls/DAB and DAPI. **(A,B)** mononuclear pollen grain, **(C,D)** binuclear pollen grain, **(E)** mature pollen grain. **(A,C,E)** Bright field images; **(B,D)** Epifluorescence images from slides **(A,C)**, respectively, showing DAPI-stained vegetative and generative nuclei. Scale bars: 20 μm.

**Figure 7:**
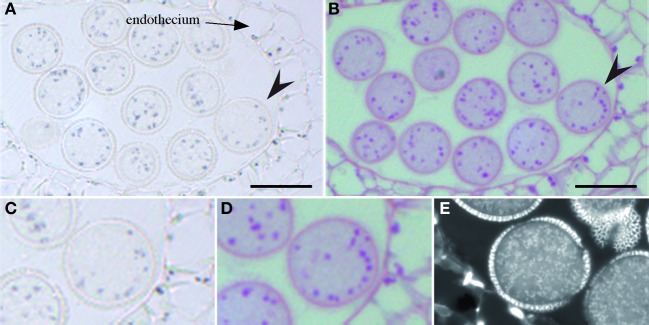
**Iron co-localizes with starch in pollen.** Serial sections of the same anther were stained with either Perls/DAB **(A,C)**, periodic acid-Schiff **(B,D)** or DiOC_6_ **(E)**. The arrow heads in **(A**,**B)** indicate the pollen grain that is magnified in **(C,D)**, respectively. Scale bars: 20 μm.

In order to investigate where iron is located in the anther, flowers were fixed and thin sections were stained with Perls/DAB. Three stages of pollen development were analyzed: monovacuolated mononucleate pollen (Figures 6A,B), binucleate pollen (Figures 6C,D), and mature pollen with degenerated tapetum (Figure 6E). The number of nuclei in the first two stages was controlled by DAPI staining (Figures 6B,D). In all three stages, iron was detected on the surface of the pollen grain, in the exine layer of the pollen coat. In addition, Fe accumulated in the cytoplasm as multiple vesicular structures, both in the pollen grains and in the different anther cell layers, including the endothecium. In all these anther cells, iron-containing compartments likely corresponded to plastids based on their number, size and position (Figure 6E). In pollen grains, after the first mitotic division, iron-stained structures tended to organize in a ring surrounding the large loosely condensed vegetative nucleus as identified by DAPI staining (Figures 6C,D). This iron organization was lost in mature pollen, showing thereafter a random distribution in the cytosol (Figure 6E). Mitochondria and amyloplasts are two potential Fe-rich organelles that were shown to aggregate around the vegetative nucleus (Yamamoto et al., 2003). In order to identify these Fe-rich bodies and potentially discriminate between mitochondria and amyloplasts, we compared the iron pattern in pollen grains with mitochondria and amyloplasts staining. Since it was technically not possible to co-stain Fe and these two organelles on the same section, two successive sections were stained with either Perls/DAB (Figure 7A) or periodic-acid/Schiff (PAS) (Figure 7B), to compare the distribution of Fe and amyloplasts stained with PAS. Another section was stained with DiOC6 for fluorescence imaging of mitochondria (Figure 7E). The Fe-rich structures fitted almost exactly with the starch-rich granules (Figures 7C, D) and this specific pattern was clearly not comparable to mitochondria, that were smaller and more numerous (Figure 7E). Taken together, these observations led us to conclude that in pollen grains Fe is highly concentrated in starch-rich structures corresponding to the amyloplasts.

## Discussion

Iron, as a cofactor for many cellular functions, is required in many sites within an organ or a cell. Studying Fe homeostasis implies, somehow, having precise information on the localization, amount and dynamics of the Fe pools in the plant. To date, such information is very fragmented and, in some cases, a matter of debate. The Perls/DAB procedure had already been described for the staining of Fe in animal tissues (Nguyen-Legros et al., 1980), but never adapted to plant material. When this procedure was adapted to plant samples, we verified that the Perls/DAB was specific for Fe ions, vs. other metals such as zinc, manganese and copper and we also demonstrated that the Perls/DAB could stain both Fe^3+^ and Fe^2+^ (Roschzttardtz et al., 2009). Potentially, Perls/DAB could thus stain most of the Fe atoms in a cell, except Fe bound to hemes since these structures do not react with these dyes (Nguyen-Legros et al., 1980). Moreover, the staining procedure directly applied to histological sections alleviated the problems of dye penetration in tissues and yielded very high-resolution images of Fe localization in cells, although the major drawback of this technique was that a portion of Fe could be lost during the fixation and dehydration steps. For instance, Fe loosely bound to soluble ligands could be drained out and some compartments such as the vacuole could appear “empty” if they contained Fe under these chemical forms (Roschzttardtz et al., 2011a). We have analyzed Fe localization in source (roots) and sink organs (leaves, flowers, and pollen) and visualized important Fe pools in several sub-cellular compartments: the cell wall, the chloroplast and a compartment in the pollen grain attributed to amyloplasts.

**Figure A1:**
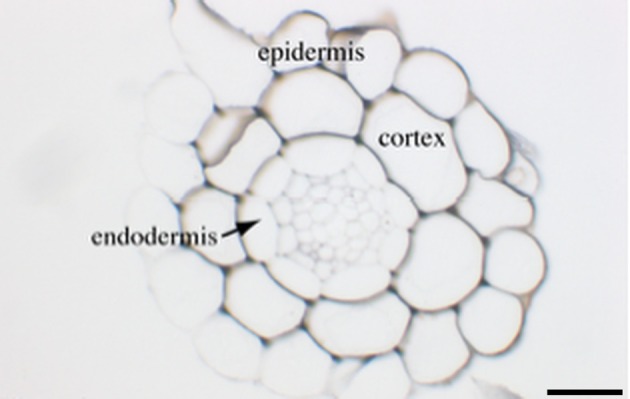
**Iron detection in Arabidopsis roots from plants grown in plate.** Three-week old wild-type plants grown in MS/2 medium supplemented with 50 μM Fe-EDTA were embedded in Technovit resin and the sections were stained with Perls/DAB. The scale bar represents 50 μm.

In roots we have shown that the vast majority of iron is located within the central cylinder since only a faint staining was visible in epidermis and cortex. Within the stele, we could also observe that Fe was principally accumulated in the cell walls, in both WT and *frd3* mutant, although in the latter case the overall Fe concentration was much higher. The role of the apoplastic compartment in Fe homeostasis and nutrition has been a matter of debate in the past. For instance, the capacity of cortical cell walls to bind Fe was estimated to reach 1000 ppm in plants cultivated in the presence of high concentrations of available (Fe-EDTA) iron (Bienfait et al., 1985) although it was later demonstrated that depending on the culture system and Fe source, the amount of apoplastic Fe pool could be overestimated (Strasser et al., 1999). The plants used in this study were grown on soil without added Fe, except in Fe excess conditions. We had chosen these growth conditions to avoid potential artifacts related to the Fe source and availability. Indeed, supplying iron as Fe-EDTA in the culture medium of axenically-grown plants, provoked an important accumulation of Fe in the apoplast of epidermal and cortical cells (Figure A1), a pattern that was not observed in the root of soil-grown plants (Figure 1). Taken together, our results establish that the apoplast can be a reservoir of Fe, mostly within the stele, and confirm the importance of this compartment in buffering Fe during the process of transport toward the aerial parts.

We have used the *frd3* mutant as a genetic model for Fe over-accumulation in roots (Green and Rogers, 2004). Like in wild type roots, most of the Fe was located in the apoplast and this pattern was particularly visible between the pericycle and the endodermis. This apoplastic accumulation included the endodermis inner half part that is internal to the casparian strip, indicating that Fe diffusion in the extracellular space is efficiently blocked by the casparian strip. A direct consequence of this observation is that Fe must reach the central cylinder via a symplastic route, which in turn suggests that an iron efflux activity is required to export iron from the endodermis and/or pericycle cells toward the apoplastic space of the central cylinder. The Ferroportin 1 (FPN1) transporter represented an appealing candidate to catalyze Fe efflux from the cells, since its expression in roots is also restricted to the central cylinder (Morrissey et al., 2009). This hypothesis was checked by testing whether the FPN1 mutation in the *frd3-7* background could decrease the apoplastic Fe accumulation in the stele. Since this was not the case, the potential role of FPN1 in the efflux of Fe toward the xylem stream in the stele was ruled out (Roschzttardtz et al., 2011b). Taken together, our data on root Fe distribution confirm and reinforce a previous report where it was proposed that the precise function of FRD3 was to facilitate Fe movement between symplastically disconnected tissues (Roschzttardtz et al., 2011b).

Within leaf cells, most of the staining was located in chloroplasts. This result, highly expected given the number of plastidial Fe-proteins, is in good agreement with earlier data that estimated that 70% of the leaf Fe lies in chloroplasts (Shikanai et al., 2003). In leaf tissues from plants grown without added iron, the staining of Fe was intense around the vascular system and very faint in mesophyll cells (Figure 2). A similar distribution has been reported in pea (Branton and Jacobson, 1962) and more recently in tobacco leaves (Takahashi et al., 2003), using ^55^Fe autoradiography and X-ray fluorescence, respectively. Fe-EDDHA supply to the plants provoked an increased abundance of Fe in most of the mesophyll cells as well as the appearance of small sized Fe rich granules in plastids. These Fe deposits were attributed to Fe-ferritin structures, based on the size, 1 μm, (Paramonova et al., 2007), the exact match with ferritin proteins detected by immunofluorescence (Figure 4) and the fact that these structures were absent in a *fer1,3,4* triple mutant devoid of ferritin proteins in leaves (Figure 3). Interestingly, ferritin-iron was more abundant in the cells associated to the vascular system. Since AtFER1, the main ferritin gene in leaves, is highly expressed in the veins (Tarantino et al., 2003), our results strongly suggest a role of this protein in buffering the excess of Fe during the process of xylem unloading. As part of the mechanism to cope with excess iron, it has recently been shown that chloroplasts can also release a fraction of the Fe pool by the efflux activity of two nicotianamine-Fe transporters, YSL4 and YSL6 (Divol et al., 2013). The mutation of these two genes results in a higher accumulation of Fe and ferritins in plastids and eventually in a higher sensitivity of the *ysl4ysl6* double mutant plants to Fe excess conditions (Divol et al., 2013). It had been previously shown that the loss of ferritin proteins in leaves did not induce a significant modification of Fe content or any macroscopic phenotype in Fe excess conditions (Ravet et al., 2009). Thanks to the Perls/DAB staining, we could show that ferritin-free chloroplasts were still able to accumulate Fe in conditions of excess (Figure 3) and, more interestingly, that this loss of ferritins provoked an important modification of the iron distribution at the cellular level with a strong accumulation in the cell walls. This redistribution of Fe outside of the cell could be the result of reduced cell uptake at the plasma membrane or the consequence of increased Fe efflux. Like in root, Ferroportin 1 could be a good candidate to fulfill this efflux function since it is expressed in the vasculature of leaves (Morrissey et al., 2009).

The vacuolar compartment is generally considered as a potential site for Fe accumulation. Such vacuolar storage was indeed found in *Arabidopsis thaliana* embryos, where Fe was detected in globoid structures inside the vacuoles (Lanquar et al., 2005) and this particular Fe pool was further located in the endodermal cell layer (Roschzttardtz et al., 2009). This vacuolar pool, remobilized by the NRAMP3 and NRAMP4 tonoplastic transporters, was shown to be an essential source of Fe during germination (Lanquar et al., 2005). Surprisingly, except in embryos, such a pool of iron was never observed in the tissues and cells studied in the present work. To some extent, this observation can be linked with the function of NRAMP3 and NRAMP4, which in the vacuoles of mesophyll cells is to export manganese and not iron (Lanquar et al., 2010).

In reproductive organs, anthers and pollen grains accumulated most of the plant stainable iron (Figure 5) indicating that the male gametophyte is a major sink of Fe. It is worth noticing that the key components of the iron uptake machinery described in roots, the ferric-chelate reductase FRO2, the ferrous iron transporter IRT1 and the citrate effluxer FRD3 are expressed in anthers (Vert et al., 2002; Connolly et al., 2003; Roschzttardtz et al., 2011b). Within the anther, iron accumulated in most of the cell layers, including the epidermis and endothecium, in structures that are compatible in size and number with plastids. Unexpectedly, the tapetum cells did not contain detectable Fe (Figures 6A,C). The absence of Fe accumulation in this important nourishing tissue could indicate that Fe is only transiently accumulated in the tapetum, before its delivery to the developing microspore cells, although it cannot be ruled out that Fe did accumulate in the tapetum in earlier stages of development. As a matter of fact, at the earliest stage considered in this study, the microspore cells (mononuclear vacuolated stage) already contained considerable amounts of Fe in the cytoplasm. The Fe-rich bodies stained in pollen grains surrounding the vegetative nucleus could correspond to mitochondria or amyloplasts, since these two organelles were shown to preferentially accumulate around the vegetative nucleus at the binuclear stage, whereas vacuoles and lipid bodies aggregate around the generative cell (Yamamoto et al., 2003). The comparison of Fe pattern with mitochondria and amyloplasts allowed to discriminate between these two organelles and established that Fe accumulated in amyloplasts. Although not demonstrated in this study, it is very likely that the Fe-rich deposits correspond to ferritins. Indeed, contrary to chloroplasts that only accumulate detectable amounts of ferritins in Fe-excess conditions, amyloplasts have been shown to contain high amounts of ferritins in physiological conditions (Yang et al., 2010). In Arabidopsis *AtFER1* is, among the four ferritin genes, the only one expressed in anthers and pollen, suggesting that this isoform could be involved in Fe storage and accumulation in pollen amyloplasts (Ravet et al., 2008). The role and importance of Fe in pollen amyloplasts remains to be demonstrated, however, it is clear that affecting long distance transport of Fe has an impact on pollen formation, development and viability. For instance, reducing NA concentration in the Arabidopsis quadruple *nas* mutant or in tobacco overexpressing a NA Amino Transferase results in a severe decrease in pollen development, viability and germination, leading to strong decrease in fertility (Takahashi et al., 2003; Schuler et al., 2012). Comparable defects have been reported in the *ysl1ysl3* double mutant (Waters et al., 2006), as well as in *frd3-7* mutant (Roschzttardtz et al., 2011b). Overall, our data have highlighted that anthers are a very important sink for iron, which is stored in amyloplats of anther tissues and pollen grains and, together with other reports on Fe transport related genes, strengthen the importance of Fe in the reproduction process.

In a previous report, we had shown that the nucleolus was an unexpected site of high Fe concentration in plant cells. Fe-rich nucleoli were observed first in pea embryos and then more generally in Arabidopsis and tomato mesophyll cells (Roschzttardtz et al., 2011a). In the present study, we have also detected Fe most likely in the nucleolus in pericycle cells in roots. The presence of Fe in the nucleolus appears thus to be a general feature of this sub-nuclear structure in plants, although the role of this specific pool of Fe is still unknown. Nevertheless, the nucleoli do not always contain high concentrations of Fe and the most striking illustration is the pollen grain. Indeed, the vegetative nucleus has a very large nucleolus that is not stained with Perls/DAB. This observation somehow suggests that Fe not strictly required as a structural element to maintain the nucleolar components together and instead, Fe may be required for the catalytic activity of the nucleolus.

### Conflict of interest statement

The authors declare that the research was conducted in the absence of any commercial or financial relationships that could be construed as a potential conflict of interest.
